# Stokes-dependent droplet collection efficiency on a NACA 0012 airfoil from droplet-informed simulations with statistical overloading

**DOI:** 10.1098/rsta.2024.0368

**Published:** 2025-07-17

**Authors:** Arash Shad, Hashnayne Ahmed, Nadim Zgheib, S. Balachandar, S A. Sherif

**Affiliations:** ^1^ Department of Mechanical and Aerospace Engineering, University of Florida, Gainesville, FL, USA; ^2^ Deptartment of Mechanical Engineering, Institute for Advanced Manufacturing,The University of Texas Rio Grande Valley, Edinburg, TX, USA

**Keywords:** ice accretion, ice physics, aircraft icing, supercooled clouds, collection efficiency, droplet impingement

## Abstract

Accurate modelling of ice accretion on aircraft wings requires analysing droplet impingement on the surface to optimize the design of ice-protection systems. We perform Euler–Lagrange simulations of a droplet-laden flow impinging on a NACA 0012 airfoil. Our study includes water droplets with eight discrete sizes ranging from 1 to 160 microns. We vary the free-stream velocity of the incoming airflow in the range 60≤U≤240 m s^−1^ and the chord length of the airfoil in the range 0.5≤c≤2 m. Due to the dilute nature of supercooled clouds, one-way coupling is used in the simulations. The effects of droplet breakup and collision are also neglected. To reduce the computational cost, we employ statistical overloading of droplets, allowing us to simulate millions of impinging droplets in a time span on the order of milliseconds. Our results show that the droplet collection efficiency, which measures the likelihood of droplet impingement on the airfoil surface, increases with droplet size and free-stream velocity but decreases with airfoil size. We demonstrate that collection efficiency, impingement velocity and impingement angle are primarily dictated by a single non-dimensional parameter, the droplet Stokes number. We also identify a critical stagnation-streamline Stokes number below which impingements do not occur and use it to estimate the minimum droplet size for impingement. In addition, we observe droplet behaviour to become Stokes number independent at large values of the Stokes number.

This article is part of the theme issue ‘Heat and mass transfer in frost and ice’.

## 1. Introduction

Modelling icing on aircraft wings is an important aspect of design to ensure flight safety, especially in scenarios where ice-protection systems may fail. Aircraft icing can result from three different processes: freezing of supercooled droplets upon surface contact, precipitation freezing on surfaces at sub-freezing temperatures and wet-snow accretion. Our focus in this paper is on the first process, namely, ice accretion due to supercooled droplet impingement. Thin cloud layers containing supercooled water droplets are commonly observed in the atmosphere and constitute nearly one-third of all mid-level clouds [1]. When a frontward surface of an aircraft passes through these clouds, the impinging droplets release their latent heat and eventually freeze on the surface resulting in ice accretion. This type of ice accretion can also be observed on wind turbines, which can lead to power losses [2]. Depending on environmental conditions, ice accretion is sometimes associated with a surface water film, which is driven by boundary-layer flow around the flying surface. After water impinges on an airfoil, droplets form a thin film that flows downstream, developing into rivulets that advance and erode from the trailing edge [3].

Accreting ice also alters surface roughness, which in turn affects the mean velocity profile of the flow over the airfoil and the convective heat transfer. Liu & Hu [4] showed that the formation of the roughness due to ice accretion enhances the convective heat transfer, with the maximum value occurring at the airfoil’s stagnation point. Bornhoft *et al.* [5] proposed a velocity transformation model for turbulent flow over rough surfaces and evaluated its accuracy in predicting aerodynamic quantities during the ice accretion process. The likelihood of ice accretion can be determined by temperature, moisture and droplet size. Normal ice accretion is observed in typical clouds that contain droplets with a median volume diameter (MVD) less than 50 µm [6]. In-flight observations have revealed a more rapid ice accretion in clouds that contain supercooled large droplets (SLDs) whose MVD exceeds 50 µm. Brown *et al.* [7] developed a phase field model for simulating the freezing of supercooled liquid droplets, demonstrating its ability to accurately model ice shapes in the SLD regime, where droplet splashing and deformation occur.

The first step in ice accretion analysis is to determine the droplet collection efficiency, which is defined as the volume fraction of droplets in the free stream that impinge on the surface. It is, therefore, essential to precisely determine the local collection efficiency to accurately predict the amount of ice accretion [8]. Published research shows that the collection efficiency decreases as droplet size decreases, eventually reaching zero below a critical droplet size [9]. The streamwise and vertical variations of cloud characteristics, including liquid water content, have an effect on the collection efficiency as do the final mass and shape of the ice layer [10]. Chang *et al.* [11] showed that by considering polydispersed droplet sizes instead of monodispersed sizes, a more realistic collection efficiency can be obtained.

A range of numerical approaches are available for simulating particle-laden flows. These include particle-resolved, Euler–Lagrange and Euler–Euler approaches. The appropriate choice of the numerical method primarily depends on the response time of particles compared to the characteristic time scale of the surrounding flow [12,13]. Euler–Lagrange and Euler–Euler approaches are widely used in icing simulations. The strengths and weaknesses of these approaches in computing the collection efficiency were assessed in [14]. The Euler–Euler approach becomes less appropriate for relatively large droplets either because the number density is too low for the validity of the continuum model or because the droplet inertia is too high to accurately represent the velocity using a single Eulerian field. For such large droplets, the Euler–Lagrange approach may be used as it allows droplets with different velocities to coexist in the same fluid grid cell [15]. Lagrangian droplet tracking algorithms have been successfully implemented to study ice accretion and calculate the collection efficiency [16,17].

Ice accretion simulations that employ the Euler–Euler approach generally lack the ability to predict a size-dependent droplet collection efficiency, impingement velocity and impingement angle. This is because an Eulerian droplet representation generally accounts for only a single droplet size, which is usually taken to be the MVD. However, it is important to account for the polydispersity of the droplet cloud to accurately evaluate the surface temperature of the accreted ice and hence the overall ice accretion process [18]. In the present study, we use the Euler–Lagrange approach with the objective of obtaining size-dependent droplet-impingement statistics including the collection efficiency, the impingement velocity and the impingement angle. As the key outcome of the paper, we show that the above characteristics are primarily controlled by a single non-dimensional parameter, namely, the droplet Stokes number. This simplified dependence enables efficient prediction of the droplet-impingement characteristics of an airfoil family under a variety of conditions that include droplet size distribution, airfoil size, free-stream velocity and ambient temperature. We also identify a critical stagnation-streamline Stokes number below which impingement does not occur and use it to estimate the minimum droplet size for impingement, representing a key contribution to advancing our understanding. Furthermore, we implement a statistical overloading approach that allows us to obtain statistically relevant data, in a computationally efficient manner.

The remainder of the paper is organized as follows: in §2, we describe the numerical approach and the parameters considered in the simulations. In §3, we present the results, where we discuss grid sensitivity in §3a and highlight the size-dependent droplet collection efficiency and its dependence on free-stream velocity and airfoil size in §3b. In §§3c–e, we discuss the droplet-impingement statistics in relation to the Stokes number. In §3f, we validate the accuracy of our results by comparing them with data available in the literature. In §3g, we examine the effect of the angle of attack on droplet-impingement statistics. Finally, conclusions are presented in §4, table 1. Table 1 lists all symbols used in the paper with their definitions.

**Table 1.** Nomenclature

latin symbols	
*c*	airfoil chord length (m)
*d*	droplet diameter (m)
**g**	gravitational acceleration vector (m s^−2^)
$\dot{m}$	mass flux per unit area (kg (m^−2^ s^−1^))
*M_p_*	droplet mass (kg)
*p*	pressure (Pa)
*R_ep_*	droplet Reynolds number
Stk	global stokes number
${\text{Stk}}_{\eta }$	stagnation streamline stokes number
*s*	curvilinear coordinate on the airfoil (m)
*t*	time (s)
u	fluid velocity vector (m s^−1^)
$u_{\eta }$	fluid velocity along the stagnation streamline (m s^−1^)
$u_{\eta }^{*}$	normalized fluid velocity along the stagnation streamline
*U*	free stream velocity (m s^−1^)
	droplet volume (m^3^)
$V_{\eta }$	droplet velocity along the stagnation streamline (m s^−1^)
**V**	droplet velocity vector (m s^−1^)
$V_{\eta }^{*}$	normalized impingement velocity
**X**	droplet position vector (m)

greek symbols	
α	angle of attack (º)
β	droplet collection efficiency
η	position on the stagnation streamline (m)
$\eta^{*}$	normalized position on the stagnation streamline
μ	dynamic viscosity (Pa s)
$\nu_{a}$	air kinematic viscosity (m^−2^ s)
$\rho_{a}$	air density (kg m^−3^)
$\rho_{W}$	water droplet density (kg m^−3^)
$\tau_{f,\eta }$	fluid time scale along the stagnation streamline (s)
$\tau_{p.\eta }$	droplet time scale along the stagnation streamline (s)
$\Theta_{\text{imp}}$	impingement angle (º)
*W*	liquid water content (g m^−3^)

## 2. Numerical set-up and methodology

**Figure 1.**
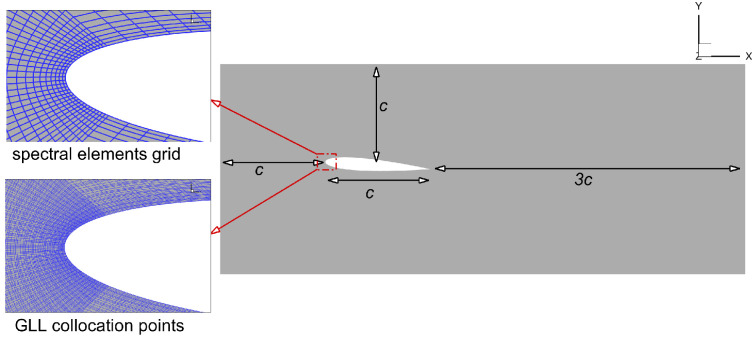
x-y slice of the computational domain along with the grid distribution. The domain extends by 0.1c along the homogeneous z direction.

The numerical set-up consists of a droplet-laden flow impinging on a NACA0012 airfoil. The airfoil has a chord length c and is placed within a cuboidal numerical domain of dimensions $L_{x}=5c$, $L_{y}=2c$ and $L_{z}=0.1c$ along the flow (x), vertical (y) and cross-flow (z) directions, respectively, as shown in figure 1.

We use an Euler–Lagrange approach, where we solve the continuity and Navier–Stokes equations for the continuous air phase and Newton’s second law for the disperse droplet phase. For the air phase, the continuity and Navier–Stokes equations are given by


(2.1) ∇.u=0,

(2.2) $$\frac{\partial u}{\partial t}+u.\nabla u=-\frac{1}{\rho }\nabla p+\nu \nabla^{2}u,$$

where u represents the three-dimensional Eulerian velocity field with components u, v and w along the x, y and z directions, respectively. The variable p represents the pressure field, t represents time and $\nu =1.3\times 10^{-5}\;{\text{m}}^{2}$ s^−1^ is the air kinematic viscosity corresponding to a free-stream temperature of $T_{a}=-5^{\circ }$C.

Regarding the disperse phase, we assume that droplets conserve mass and remain in thermal equilibrium with the surrounding air. Consequently, the governing equations of the position, X, and velocity, V, of the $p^{th}$ droplet are


(2.3) $$\frac{dX_{p}}{dt}=V_{p},$$

(2.4) $$M_{p}\frac{dV_{p}}{dt}=3\pi \mu d(u_{p}-V_{p})\phi (Re_{p})+V_{p}(\rho_{w}-\rho_{a})g.$$

Here $M_{p}=\rho_{w}V_{p}$ represents the mass of the $p^{th}$ droplet of diameter d and volume $V_{p}=\pi d^{3}/6$, and μ represents the air dynamic viscosity. The density of all droplets is taken as $\rho_{w}=1000\;{\text{kg m}}^{-3}$, and air density is $\rho_{a}=1.3\;{\text{kg m}}^{-3}$, which corresponds to a free-stream temperature of $T_{a}=-5^{\circ }$C. The first term on the right-hand side of equation (2.4) represents the quasi-steady drag force with a finite Reynolds number correction, where $u_{p}$ is the fluid velocity at the droplet location. The second term represents the gravitational force. The correction function ϕ accounts for the finite Reynolds number effect and is given by


(2.5) $$\phi (Re_{p})=1+0.15Re_{p}^{0.687},$$

where the Reynolds number of the $p^{th}$ droplet is


(2.6) $$Re_{p}=|u_{p}-V_{p}|d/\nu .$$

The intent of the present set-up is to simulate the impingement of supercooled water droplets onto the NACA 0012 airfoil. In the context of atmospheric icing, the volume fraction of supercooled droplets in a cloud is typically very low: on the order of $10^{-6}$ [19]. As such, the droplet-laden airflow can be treated as a dilute gas-droplet system. Consequently, droplet–droplet interactions and the back-coupling from the discrete droplet phase to the continuous air phase can be ignored. Therefore, the current simulations assume one-way coupling such that the airflow influences the droplet velocity and position, but the droplets do not influence the airflow. In addition, we ignore the effects of droplet breakup, splashing and coalescence.

In a typical cloud, ice accretion occurs on a time scale of several minutes. Correspondingly, in the Euler–Lagrange simulations, if we employ the droplet volume fraction to be the same as in an actual cloud, ice accretion must be simulated for a time duration on the order of minutes to obtain statistically converged results for the collection efficiency of the various droplet sizes. For numerical stability, the time step is typically limited to be of the order of microseconds, and therefore, simulating several minutes of ice accretion is computationally expensive. To overcome this problem, we statistically overload the cloud by increasing the droplet volume fraction by a factor of approximately 200 to a value of approximately $2\times 10^{-4}$. Theis statistical overloading allows us to obtain statistically converged results at a fraction of the computational cost. We note that statistical overloading is only possible due to the one-way coupling nature of the problem. Statistical overloading has been used in other problems where the one-way coupled Euler–Lagrange methodology is employed to obtain statistically relevant results [20,21].

Since the spanwise flow dependence is weak, the simulations employ a narrow spanwise domain of size 0.1c with periodic conditions along the z direction. A uniform inflow, u=(U,0,0), is applied at x=0, while outflow conditions are set at x=5c. A free-slip condition with ∂u/∂y=0 is enforced at y=±c. Finally, a no-slip, no-penetration condition, u=(0,0,0), is applied on the airfoil surface. The constant inflow velocity U is chosen between 60 and 240 m s^−1^ in the different simulations, as listed in table 2. The chord length c is chosen to be between 0.5 and 2 m in the different simulations. This results in a Reynolds number, based on free-stream velocity and chord length, ranging from $4.3\times 10^{6}$ to $1.7\times 10^{7}$ as listed in table 2. These conditions are chosen to align with the typical range of values reported in existing studies [17,19] and to ensure comparable Stokes numbers across the simulations.

**Table 2.** Details of the simulations conducted showing inlet velocity (U), chord length (c), angle of attack (α), polynomial order (N) and Reynolds number (Re=Uc/ν). All simulations considered droplet diameters (d) of 1, 5, 13, 18, 29, 48, 97 and 160 µm.

case name	U (m s^−1^)	c (m)	α ( ${,}^{\circ }$ )	N	Re
S1	60	1.0	4	6	$4.3\times 10^{6}$
S2	120	1.0	4	6	$8.6\times 10^{6}$
S3	240	1.0	4	6	$1.7\times 10^{7}$
S4	120	0.5	4	6	$4.3\times 10^{6}$
S5	120	2.0	4	6	$1.7\times 10^{7}$
S6	120	1.0	6	6	$8.6\times 10^{6}$
S7	60	1.0	4	8	$4.3\times 10^{6}$
S8	60	1.0	4	12	$4.3\times 10^{6}$

To achieve statistically converged results over a range of droplet sizes, we need to inject a large sample of droplets of varying sizes. In all the simulations, we consider eight discrete droplet diameters, d=1, 5, 13, 18, 29, 48, 97 and 160 microns, as detailed in table 2. This range was chosen to encompass a broad spectrum of droplet sizes, from small droplets to those characteristic of the SLD regime, similar to freezing drizzle. At each time step, we inject a total of 400 droplets, corresponding to a rate of 50 droplets per size per time step. Droplets are injected with uniform random probability into a cuboidal region of dimensions $l_{x}\times l_{y}\times l_{z}=0.03c\times 0.08c\times 0.1c$ at a distance of approximately 0.3c from the leading edge of the airfoil. The injected droplets span the full width of the domain but are confined to a rectangular area ($A_{\infty }=l_{y}\times l_{z}$) in the y-z plane, extending over a distance $l_{x}$ along the flow direction. The magnitude of $l_{y}$ is chosen to sufficiently exceed the airfoil thickness in the vertical y direction. This strategy is adopted to optimize computational cost since the vast majority of droplets located at y<0.01c or y>0.1c will not impinge on the airfoil and thus represent a computational loss. The injection velocity of droplets matches the inlet velocity, given by V=(U,0,0). The prescribed injection rate of 50 droplets per time step per size results in a total of one million droplets per size over the entire duration of the simulation, amounting to eight million droplets overall. Such a large sample size ensures the convergence of statistically significant results.

Different injection rates were tested, and we observed that increasing the injection rate beyond 50 droplets per time step per size does not affect the collection efficiency or other statistics reported in the study. Therefore, we adopted the aforementioned rate of droplet injection. A droplet with a diameter d is considered to have impinged on the airfoil if the distance between the droplet’s centre and the airfoil surface is 0.5d or less. In other words, if the droplet makes contact with the airfoil, it is removed from the computational domain and considered to have impinged at the point of contact.

The present simulations are conducted using the massively parallel, spectral element solver Nek5000 [22,23], which is well suited for meshing the complex airfoil geometry. Within each element, the Eulerian fields are represented by a polynomial of order N through polynomial interpolants located at Gauss–Lobatto–Legendre (GLL) points, with *N* + 1 GLL points along each element direction. As for time integration, we use the third-order backward difference scheme. The mesh is constructed to conform to the airfoil shape and then extruded uniformly in the z direction. A mesh consisting of 17 400 elements is used across all simulations. The ordinary differential equations (ODEs) for tracking droplet trajectories and velocities are solved using ppiclF, a parallel particle-in-cell library in FORTRAN [24,25].

### (a) Droplet trajectory and impingement

A total of eight simulations whose details are listed in table 2 were conducted. For each simulation, we specify the free-stream velocity U, chord length c, angle of attack α, polynomial order N and the resulting Reynolds number based on inlet velocity and chord length. The primary objective of these simulations is to collect data on the droplet collection efficiency (β), droplet-impingement velocity ($V_{I}$) and the impingement angle at the time of impingement (Θ).

**Figure 2.**
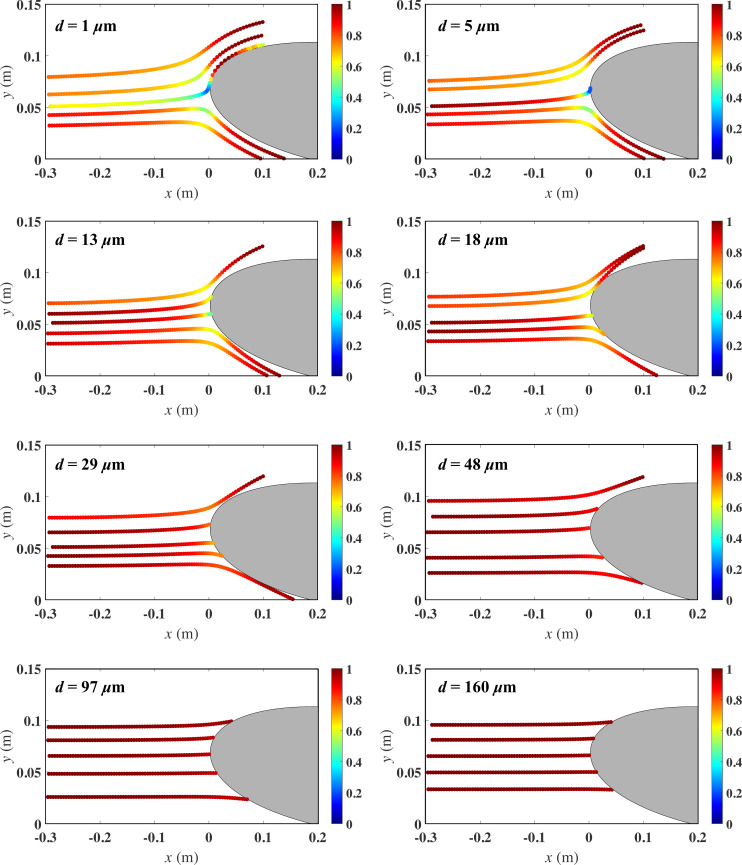
Droplet trajectories for the different droplet diameters from case S2 coloured by the local velocity normalized by the free-stream velocity U. The smallest droplets follow the flow faithfully and are extremely unlikely to impinge onto the airfoil, whereas the largest droplets have near ballistic trajectories, whereby they are minimally affected by the airflow.

To provide a visual description of the nature of the present simulations, we show in figure 2 droplet trajectories corresponding to all eight droplet sizes from case S2. Each of the eight panels of figure 2 corresponds to a particular droplet size as indicated in each respective panel. The droplet trajectories are shown starting from the injection point located upstream at x≈−0.3c, where they are injected at the free-stream velocity U. The trajectories are colour-coded in relation to the local droplet velocity, V, normalized by the free-stream velocity U. It is clear from the figure that droplet behaviour varies significantly with droplet size. For instance, the smallest micron-size droplets (*d* = 1 µm) are observed to follow the airflow faithfully exhibiting relatively large variations in droplet velocity magnitude and direction along their trajectories. On the other hand, for the largest droplets (*d* = 160 µm), the droplet velocity magnitude remains close to the free-stream value. Though the trajectories remain largely horizontal, some deflection is observable close to the airfoil surface, even for such large droplets. It should be noted that the effect of droplet deformation and potential breakup is ignored. On the other hand, the behaviour of intermediate droplet sizes falls within the bounds of the aforementioned limiting cases of very small and very large droplets.

### (b) Droplet collection efficiency

The droplet collection efficiency, β, is defined as the ratio of the local mass flux of impinged droplets at the airfoil surface to the mass flux of droplets in the free stream, as suggested in [26]. The mass flux of droplets per unit area of droplet injection in the free stream (${\dot{m}}_{\infty }$) can be expressed as the product of the free-stream liquid water content ($\omega_{\infty }$) and the free-stream velocity (U)


(2.7) $$\begin{array}{lcr} & {\dot{m}}_{\infty }=\omega_{\infty }U\;. & \end{array}$$

The droplet collection efficiency at any location on the airfoil surface can in turn be defined as


(2.8) $$\begin{array}{lcr} & \beta =\frac{{\dot{m}}_{\text{coll}}}{{\dot{m}}_{\infty }}\;. & \end{array}$$

**Figure 3.**
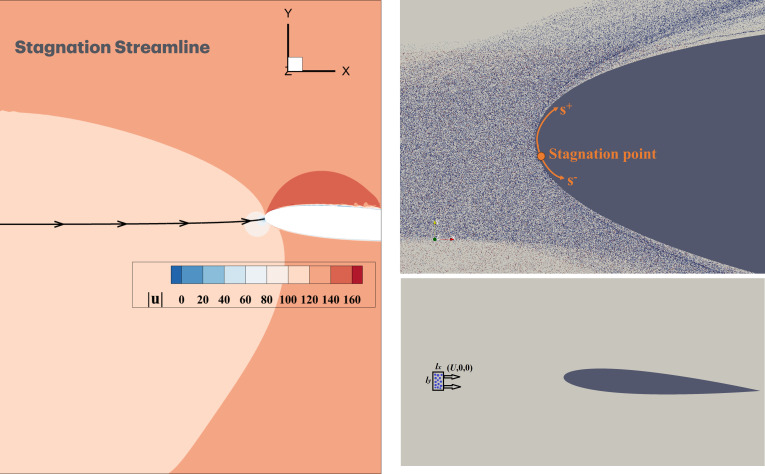
(Left) Stagnation streamline. (Top right) Droplet distribution in the domain at a particular instant. (Bottom right) Injected droplets within the cuboidal location for droplet injection ($l_{x}\times l_{y}\times l_{z}$).

Here, ${\dot{m}}_{\text{coll}}$ denotes the mass flux of droplets impinging per unit area of the airfoil surface. The value of ${\dot{m}}_{\text{coll}}$ is expected to vary along the surface of the airfoil. Typically, ${\dot{m}}_{\text{coll}}$ is highest near the stagnation point and decreases progressively as the curvilinear distance from the stagnation point along the airfoil surface increases, i.e. s becoming increasingly positive (corresponding to the upper surface of the airfoil) or negative (corresponding to the lower surface of the airfoil) as depicted in figure 3. In addition, ${\dot{m}}_{\text{coll}}$ will also depend on droplet size, generally increasing as the droplet size increases.

We note here that, due to the stochastic nature of the droplet injection process, ${\dot{m}}_{\infty }$, ${\dot{m}}_{\text{coll}}$ and β are stochastic quantities exhibiting temporal variation. However, by averaging over a long enough time period ($\Delta t_{\text{coll}}$), the mass fluxes and, consequently, the collection efficiency will converge to a mean value. The converged mean value of the collection efficiency ⟨β⟩ is expressed as


(2.9) $$\langle \beta \rangle =\frac{\left\langle {\dot{m}}_{\text{coll}}\right\rangle }{\langle \omega_{\infty }\rangle \;U},$$

where the angle brackets indicate time averaging over the time period $\Delta t_{\text{coll}}$.

## 3. Results and discussions

### (a) Grid sensitivity analysis and β dependence on droplet diameter

**Figure 4.**
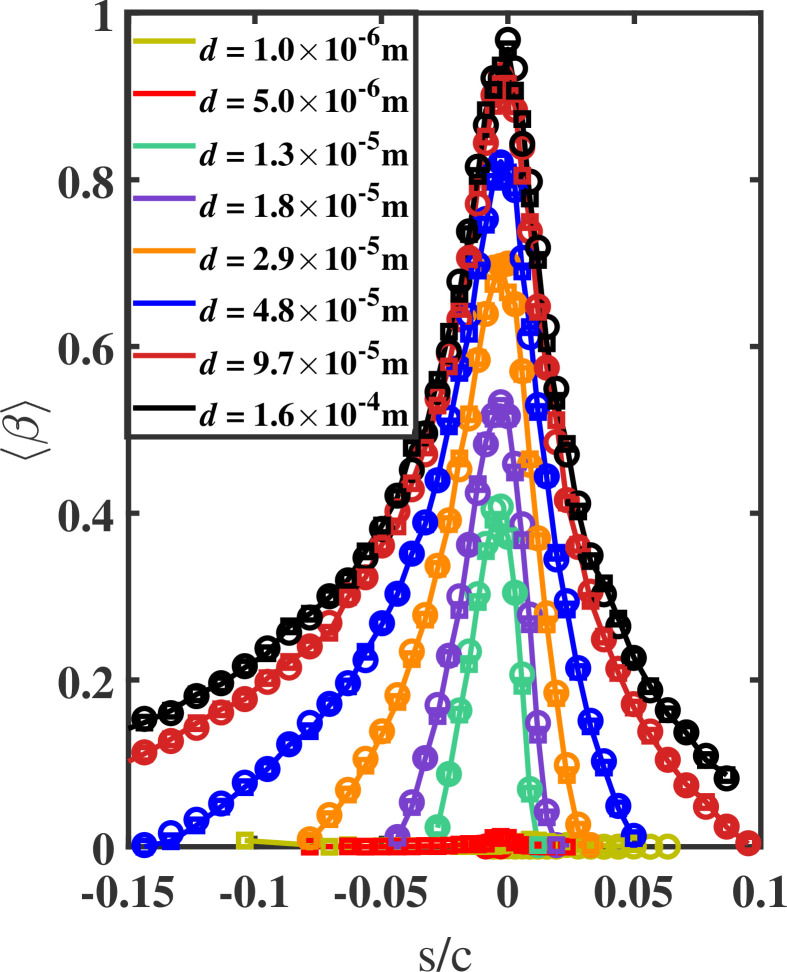
Collection efficiency versus the scaled curvilinear position (s/c) on the airfoil surface for all droplet sizes from cases S1 (lines), S7 (circle markers) and S8 (square markers). Results indicate that N=6 is a suitable polynomial order that provides grid-independent results for the required statistics.

In figure 4, we plot ⟨β⟩ against the scaled curvilinear distance s/c, where s=0 denotes the location of the stagnation point. To obtain ⟨β⟩, the airfoil surface is discretized into a rectangular mesh in the s-z plane conforming to the shape of the airfoil. Each rectangle has a cross-sectional area of Δs×Δz, where 0.0002c≤Δs≤0.01c and Δz=c/3. In addition, due to the homogeneous nature of the spanwise z direction, all quantities in the manuscript, including ⟨β⟩, were span-averaged. The value of Δs was chosen small enough to extract the spatial variation, especially near the leading edge. The quantity ⟨β⟩ is plotted for each of the eight droplet sizes from simulations S1, S7 and S8. As listed in table 2, the three simulations differ only in the polynomial order N, with N=6 for S1, N=8 for S7 and N=12 for S8. For the 1- and 5-micron droplets, the maximum value of ⟨β⟩ reaches approximately 2% near the stagnation point and remains at near-zero values elsewhere. This trend is consistent across all three cases considered, indicating that under the present conditions of *U* = 60 m s^−1^ and c=1m, small micron-sized droplets are generally unlikely to collide with the airfoil surface. We observe excellent agreement between the three cases across all droplet sizes, with the $L_{1}$ norm of the difference consistently remaining below 4%. The L1 norm is a mathematical concept that calculates the size of a vector by summing the absolute values of all its components. We conclude that the results are grid-independent for a grid resolution of N≥6. Consequently, we adopt a polynomial order of N=6 for all other simulations, as listed in table 2.

### (b) β dependence on free-stream velocity and airfoil size

It is clear from figure 4 that ⟨β⟩ varies with droplet size. In fact, all things being equal, ⟨β⟩ is expected to increase with droplet diameter, d, and this behaviour is confirmed in figure 4. In addition to d, ⟨β⟩ is expected to depend on the free-stream velocity U and the airfoil size c. The higher the free-stream velocity, the greater the droplet inertia relative to the flow, leading to a relative increase in the droplet response time scale. Consequently, droplets require more time to react to flow changes, especially near the airfoil leading edge where streamlines experience the most significant variations. Therefore, as the free-stream velocity increases, the likelihood of droplets colliding with the airfoil also increases. On the other hand, the larger the airfoil size, the larger the distance upstream of the airfoil where the flow begins to adjust to the presence of the airfoil, and streamlines begin to deviate from their straight path. For the same free-stream velocity, the larger distance provides droplets with a longer time to react and adjust to the varying flow conditions. Consequently, the likelihood of droplets colliding with the airfoil decreases with increasing airfoil size.

**Figure 5.**
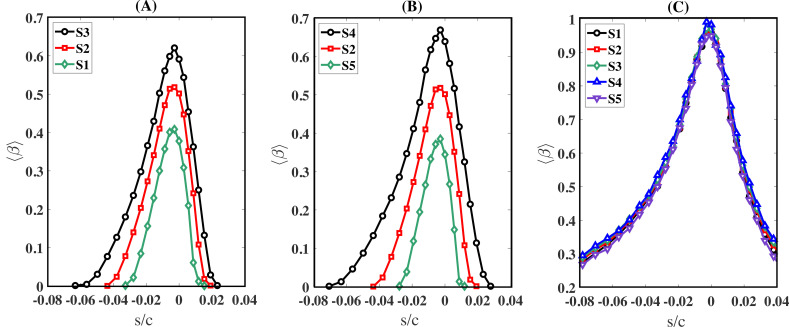
Droplet collection efficiency versus the scaled curvilinear position (s/c) on the airfoil surface for two specific droplet sizes from cases S1 to S5. Panels A and B correspond to 13-micron droplets, and panel C corresponds to 160-micron droplets.

Figure 5 displays the droplet collection efficiency, ⟨β⟩, for three different simulations with varying free-stream velocities and chord lengths. Panel A shows ⟨β⟩ for droplets with a diameter of *d* = 13 µm from simulations S1, S2 and S3. The only difference between these simulations is the free-stream velocity, U, which is set to 60, 120 and 240 m s^−1^ for S1, S2 and S3, respectively. As illustrated in the figure, the magnitude and extent of ⟨β⟩ increase progressively. For instance, the maximum value of ⟨β⟩ rises from approximately 0.41 for S1 to 0.52 for S2 and further to 0.62 for S3. Furthermore, the surface area affected by droplet impingement expands. For S1, droplets impinge over the curvilinear range of approximately −0.03≲s/c≲0.01, whereas for S3, this range extends to approximately −0.06≲s/c≲0.03.

Panel B shows ⟨β⟩ again for droplets with a diameter of *d* = 13 µm, obtained from simulations S2, S4 and S5. The only difference between these simulations is the chord length c, which determines the airfoil size. Specifically, c is set to 1 m for S2, 0.5 m for S4 and 2 m for S5. As shown in the figure, both the magnitude and extent of ⟨β⟩ decrease progressively. For example, the maximum value of ⟨β⟩ decreases from approximately 0.68 in S4 to 0.39 in S5. In addition, the area affected by droplet impingement contracts as well. In S4, droplets impinge over the curvilinear range of approximately −0.07≲s/c≲0.03, whereas in S5, this range narrows to approximately −0.03≲s/c≲0.01.

Panel C shows ⟨β⟩ for the largest droplets considered, specifically those with a diameter of *d* = 160 µm, obtained from simulations S1, S2, S3, S4 and S5. Note that, ⟨β⟩ with a maximum value of approximately 1 is unchanged across all five simulations, in contrast with the previous behaviour observed in panels A and B. The reason for this behaviour is that for very large droplets, the droplet time scale, which determines the relaxation time droplets need to adjust to changing flow conditions, is quite large and as a result, their trajectory remains ballistic [27], with their motion minimally affected by the flow over short time scales.

### (c) Stokes number scaling

#### (i) Flow and droplet time scales

Although the droplet collection efficiency (⟨β⟩) has been shown to vary with the droplet diameter (d), free-stream velocity magnitude (U) and airfoil size as dictated by its chord length (c), it is not the individual contributions of these parameters that govern the variations in ⟨β⟩. Instead, it is their combined effect, encapsulated by the Stokes number, which dictates these variations. In droplet-laden flows, the Stokes number, Stk, is defined as the ratio of the droplet time scale $\tau_{p}$ to the flow time scale $\tau_{f}$ [28]. In the present context of a stagnation-point flow, we consider the flow time scale along the stagnation streamline to vary as [29]


(3.1) $$\tau_{f,\eta }=\frac{1}{du_{\eta }/d\eta }.$$

Here, η denotes the curvilinear coordinate along the stagnation streamline, while $u_{\eta }$ represents the corresponding velocity magnitude along this streamline. The flow time scale, as defined in equation (3.1), is thus inversely proportional to the rate of change of the velocity magnitude along the streamline. The droplet time scale may then be approximated by solving the below system of ODEs along the stagnation streamline [29] (ignoring the effect of streamline curvature)


(3.2) $$\frac{d\eta }{dt}=V_{\eta },\;\frac{dV_{\eta }}{dt}=\frac{u_{\eta }-V_{\eta }}{\tau_{p,\eta }},$$

subject to the initial condition of η(t=0)=0 and $V_{\eta }(t=0)=u_{\eta }=0.99U$. In equation (3.2), $V_{\eta }$ and $u_{\eta }$ represent the droplet and fluid velocity magnitudes along the stagnation streamline, respectively, where the droplet time scale is expressed as [29]


(3.3) $$\tau_{p,\eta }=\frac{\rho_{w}d^{2}}{18\rho_{a}\nu_{a}\phi ({\text{Re}}_{p})}$$

and where the finite Reynolds correction function $\phi ({\text{Re}}_{p})$ was defined earlier in equation (2.5). Both $\tau_{f,\eta }$ and $\tau_{p,\eta }$ vary along the stagnation streamline and, as such, their ratio $Stk_{\eta }=\tau_{p,\eta }/\tau_{f,\eta }$ will also vary.

**Figure 6.**
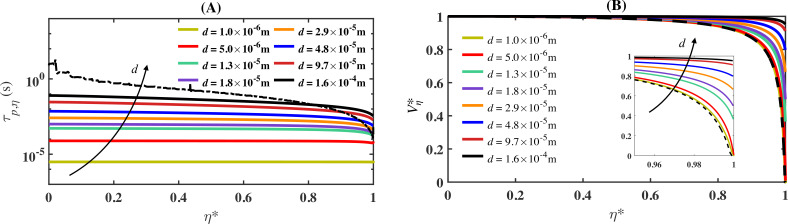
Comparison of the droplet time scale ($\tau_{p,\eta }$) and the normalized droplet velocity ($V_{\eta }^{*}$) along the stagnation streamline for various droplet sizes as a function of the normalized position $\eta^{*}=\eta /\eta_{\text{stag}}$ from S2. The black dashed line in panels A and B represents the fluid time scale ($\tau_{f,\eta }$) and the normalized fluid velocity ($u_{\eta }^{*}$), respectively, along the stagnation streamline.

Figure 6 shows $\tau_{p,\eta }$ along with $u_{\eta }$ and $V_{\eta }/U$ for all droplet sizes from case S2. Panels A and B depict these variables versus the normalized position along the stagnation streamline, $\eta^{*}=\eta /\eta_{\text{stag}}$, where $\eta_{\text{stag}}$ represents the value of η at the stagnation point. We observe the expected behaviour for all four variables considered. For example, in panel A, $\tau_{f,\eta }$ is highest at $\eta^{*}=0$, the farthest upstream point, and decreases along the streamline, reaching its minimum at the stagnation point where $\eta^{*}=1$. Notably, the rate of decrease intensifies along the streamline. This is anticipated because, far upstream, the rate of velocity change is minimal, while near the airfoil, the velocity drops relatively sharply. On the other hand, $\tau_{p,\eta }$ is shown to increase monotonically with droplet diameter for any location along the streamline. Also, similar to $\tau_{f,\eta }$*,* the quantity $\tau_{p,\eta }$ decreases along the streamline at a rate that also intensifies closer to the stagnation point. The relative value of $\tau_{p,\eta }$ to $\tau_{f,\eta }$ dictates how faithfully a droplet would follow the flow (whether or not it would impinge on the surface) and the magnitude of the impingement velocity.

As shown in panel B, the 1-micron droplet velocity follows closely the fluid velocity of the stagnation streamline, with the latter shown as a dashed black line. As droplet size increases, so does droplet inertia. As a result, larger droplets require a longer time to adjust to changes in flow velocity, which translates to larger differences between the droplet and fluid velocities. Moreover, as the difference increases, the likelihood of impingement (and impingement velocity magnitude) also increases, and it does so monotonically. For instance, for the 160-micron droplets, the impingement velocity at the stagnation point is approximately 96% of the free-stream velocity, i.e. ${\text{V}}_{\eta }\approx 0.96U$. Whereas for the 13-micron droplets, the impingement velocity decreases to slightly less than 40% of the free-stream velocity.

#### (ii) Critical Stokes number

**Figure 7.**
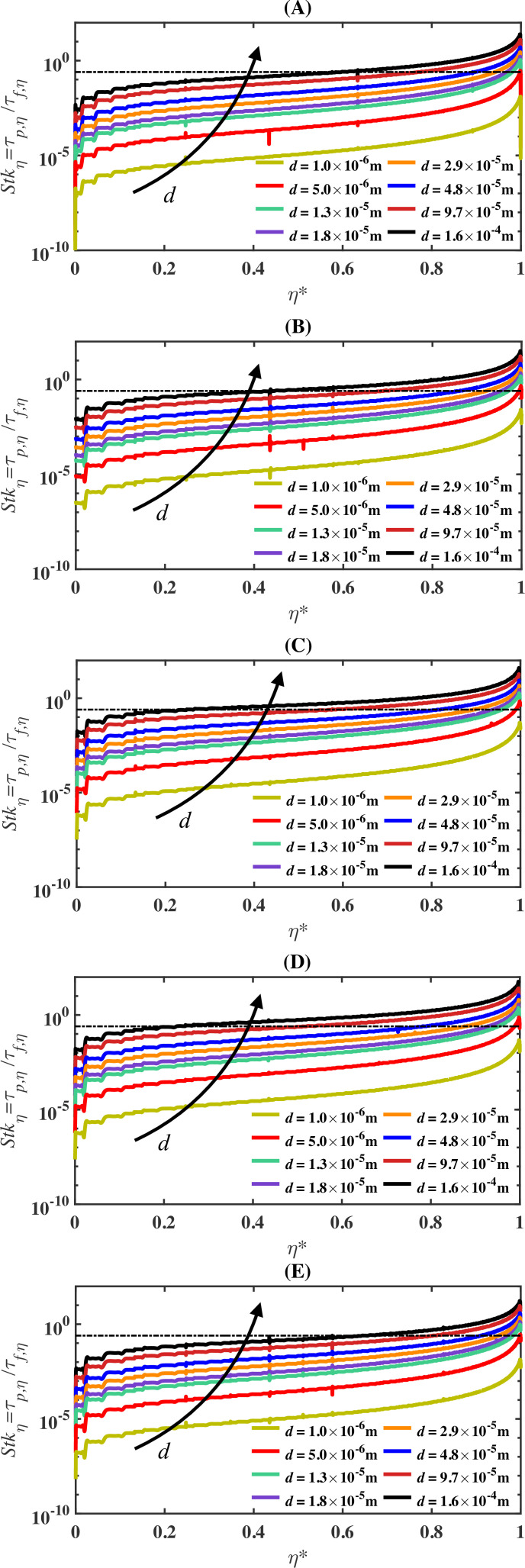
Stagnation-streamline Stokes number ${\text{Stk}}_{\eta }$ versus the scaled stagnation-streamline location $\eta^{*}=\eta /\eta_{\text{stag}}$ for case S2 (panel (A)). Panels (B) to (E) show the same information for cases S1, S3, S4 and S5, respectively. The dash-dotted black line in each panel corresponds to the critical Stokes number of ${\text{Stk}}_{\eta ,cr}=0.25$.

Figure 7 depicts the stagnation-streamline Stokes number, ${\text{Stk}}_{\eta }$, versus the scaled stagnation coordinate $\eta^{*}$ for cases S1 to S5 and for all droplet diameters. In the case of stagnation-point flows, Ferry *et al.* [29] argued that droplets would impinge on the surface provided the stagnation-streamline Stokes number exceeds a critical value of ${\text{Stk}}_{\eta ,cr}=0.25$. This threshold value is inserted into figure 7 as a dash-dotted black line. Far upstream, i.e. for small values of $\eta^{*}$, the flow time scale is sufficiently larger than the time scale of all the droplet sizes, such that ${\text{Stk}}_{\eta }<{\text{Stk}}_{\eta ,cr}$. However, depending on the droplet size, the critical value could be exceeded along the streamline. For instance, in the case of S2 (depicted in panel (B) of figure 7) and for the 160-micron droplets, the location where ${\text{Stk}}_{\eta }$ exceeds the critical value (${\text{Stk}}_{\eta ,cr}$) is approximately $\eta^{*}=0.45$, whereas for *d* = 29 µm, the critical Stokes value is exceeded approximately $\eta^{*}=0.9$. On the other hand, for the 1-micron droplet, the critical Stokes value is never exceeded. As indicated earlier, the Stokes number is not solely a function of droplet diameter but also depends on other parameters including airfoil size and free-stream velocity. In fact, for cases S1 to S5, the Stokes number for the 1-micron droplet never exceeds the threshold value. Moreover, the collection efficiency of droplets whose stagnation-streamline Stokes number remains below the threshold value is near zero over the entire airfoil.

**Figure 8.**
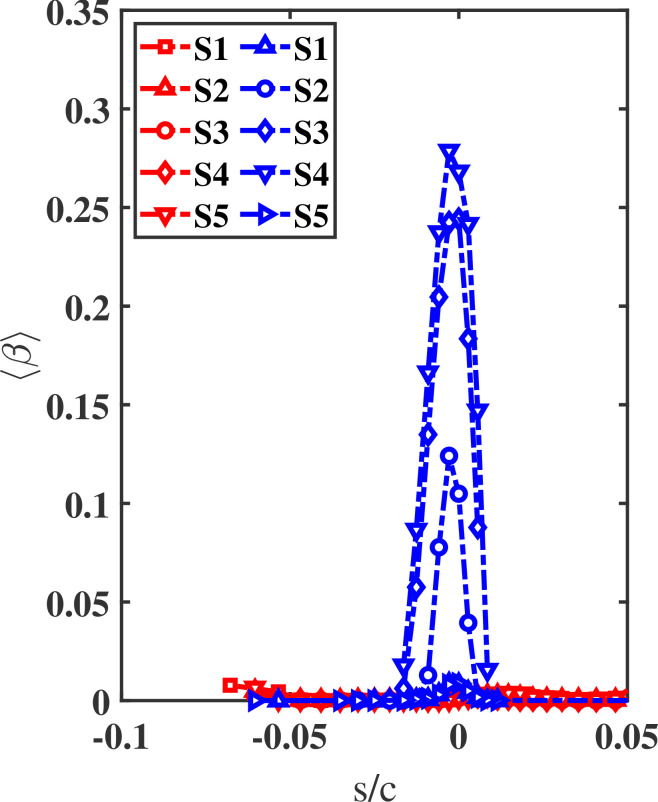
Collection efficiency (⟨β⟩) versus airfoil surface location s/c for the 1- and 5-micron droplets (red and blue colours, respectively) from cases S1, to S5. ⟨β⟩≈0 when the stagnation-streamline Stokes number remains below the threshold value of 0.25.

Figure 8 shows ⟨β⟩ versus s/c for the 1- and 5-micron droplets from cases S1 to S5. The plot highlights the effect of the critical Stokes number in predicting whether or not droplets impinge on the surface. Results are in agreement with [12,29] in that only those droplets whose stagnation-streamline Stokes number exceeds the critical value impinge on the airfoil. For instance, the plot confirms that the 1-micron droplets from all cases in addition to the 5-micron droplets from S1 and S5 have a near-zero collection efficiency. This is in line with the fact that the stagnation-streamline Stokes number remains below the critical value.

In summary, for an airfoil at any angle of attack, potential flow around the airfoil can be calculated either analytically or computationally to obtain the normal velocity gradient ∂u/∂η at the stagnation point. In terms of this gradient, a minimum droplet size for collision can be estimated as


(3.4) $$d_{\text{min}}=\sqrt{\frac{9}{2}\frac{\rho_{a}\nu_{a}}{\rho_{w}}\frac{1}{du/d\eta }}.$$

Incoming droplets that are smaller than this minimum diameter will avoid collision. The fact that very small droplets escape from impacting the airfoil surface has been recognized in experiments. However, a precise criterion for the minimum droplet size for impaction has hitherto not been established, partly because past investigations have generally considered a spectrum of droplet sizes, typical of atmospheric clouds and focused on net the collection efficiency, instead of size- or Stokes number-dependent collection efficiency. Future experimental validation of the above estimation of the minimum droplet size for collision is needed.

### (d) Droplet-impingement angle and velocity

**Figure 9.**
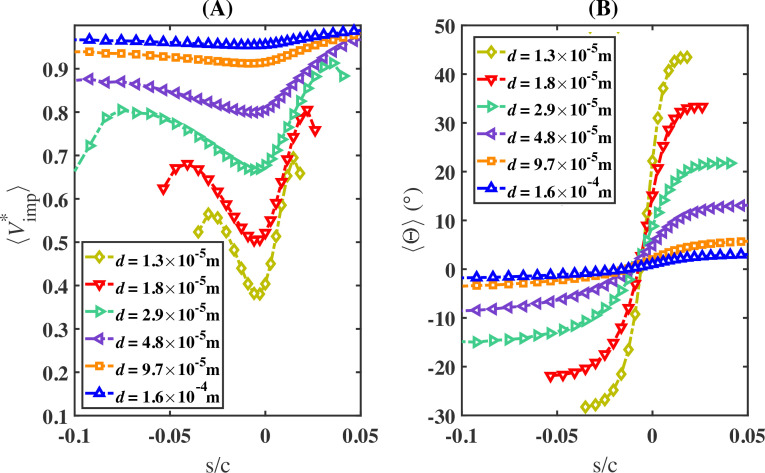
Normalized impingement velocity magnitude ($\left\langle V_{\text{imp}}^{*}\right\rangle$) and impingement angle (⟨Θ⟩) obtained from case S2 versus the scaled curvilinear position (s/c) on the airfoil surface.

The impingement angle and velocity can vary significantly across the airfoil surface. In figure 9, panel (A) shows the average impingement velocity magnitude, scaled by the free-stream velocity ($\left\langle V_{\text{imp}}^{*}\right\rangle$), plotted against s/c, while panel (B) shows the average impingement angle (⟨Θ⟩) also plotted against s/c. Both results are obtained from case S2. Recall that the airfoil surface was discretized into rectangular cells to compute ⟨β⟩, and the same procedure is used to compute $\left\langle V_{\text{imp}}^{*}\right\rangle$ and ⟨Θ⟩. Results are presented only in regions where $\langle \beta_{i}\rangle >0.05$. As shown in panel A, we observe that $\left\langle V_{\text{imp}}^{*}\right\rangle$ increases with droplet diameter and exhibits greater spatial variation along s as droplet size decreases. For instance, $\left\langle V_{\text{imp}}^{*}\right\rangle$ for the 160-micron droplets ranges from 0.95 to 0.99, whereas for the 13-micron droplets, it spans a broader range from 0.38 to 0.69. In addition, while the collection efficiency (⟨β⟩) is highest near the stagnation point, $\left\langle V_{\text{imp}}^{*}\right\rangle$ reaches its lowest value at this point for all droplet sizes. Furthermore, $\left\langle V_{\text{imp}}^{*}\right\rangle$ increases away from the stagnation point on both sides of the airfoil, reaching its maximum value on the upper side (i.e. for s>0).

The average impingement angle (⟨Θ⟩), shown in panel B, is computed using cell-average values, similar to the computation of $\left\langle V_{\text{imp}}^{*}\right\rangle$. The impingement angle ⟨Θ⟩ is measured counter-clockwise relative to the horizontal x-axis. Like $\left\langle V_{\text{imp}}^{*}\right\rangle$, ⟨Θ⟩ exhibits a monotonic trend, with its range over s varying according to droplet diameter and increasing as droplet diameter decreases. For instance, ⟨Θ⟩ for the 160-micron droplets ranges from $-1.8^{\circ }$ to $3.2^{\circ }$, whereas for the 13-micron droplets, it spans a broader range from $-28^{\circ }$ to $43^{\circ }$. In addition, ⟨Θ⟩ is observed to increase monotonically with s and to exhibit an inflexion point with a near-zero value in the vicinity of the stagnation point towards the nose of the airfoil. We note here that, while the results in figure 9 are only reported for case S2, similar behaviour is observed across all other cases for both $\left\langle V_{\text{imp}}^{*}\right\rangle$ and ⟨Θ⟩. In fact, we observe both $\left\langle V_{\text{imp}}^{*}\right\rangle$ and ⟨Θ⟩ as well as the collection efficiency ⟨β⟩ to exhibit a unified behaviour with the Stokes number, as will be shown in §3e.

### (e) Unified behaviour of ⟨β⟩, $\left\langle V_{\text{imp}}^{*}\right\rangle$ and ⟨Θ⟩ with the Stokes number

The stagnation-streamline Stokes number (${\text{Stk}}_{\eta }$) is useful for assessing the likelihood of droplet impingement. For the present discussion, we define a global Stokes number Stk as


(3.5) $$\text{Stk}=\frac{\rho_{w}d^{2}/18\rho_{a}\nu_{a}}{c/U}.$$

In this definition, the flow time scale $\tau_{f}$ is taken as c/U, which is a constant for each simulation. The droplet time scale $\tau_{p}$ is taken to be time- and space-invariant (i.e. also a constant), given by $\rho_{w}d^{2}/18\rho_{a}\nu_{a}$. This definition provides a unique Stokes number for each droplet in a particular case.

**Figure 10.**
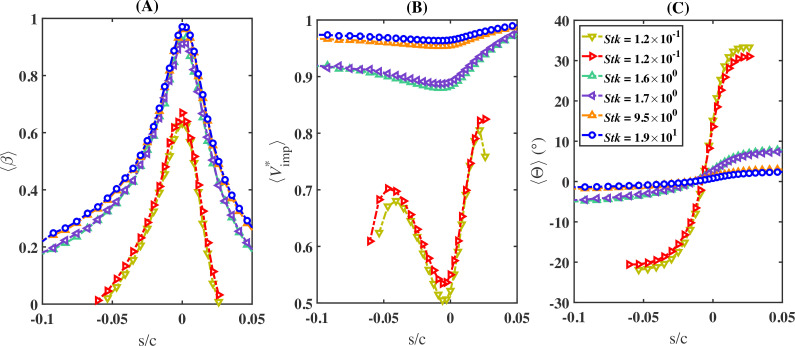
Variations in (A) collection efficiency (⟨β⟩), (B) normalized impingement velocity ($\left\langle V_{\text{imp}}^{*}\right\rangle$) and (C) impingement angle (⟨Θ⟩) as functions of the Stokes number versus the scaled curvilinear position (*s*/*c*) on the airfoil surface.

Figure 10 shows the unified behaviour of ⟨β⟩ (panel A), $\left\langle V_{\text{imp}}^{*}\right\rangle$ (panel B) and ⟨Θ⟩ (panel C) for a varying Stokes number Stk. Each panel contains six curves corresponding to different droplet sizes from the various cases considered in table 2. In particular, following the same order in the legend in panel C, the selected data correspond to droplets of diameter *d* = 18 µm from S2, *d* = 13 µm from S4, *d* = 97 µm from S1, *d* = 48 µm from S4, *d* = 160 µm from S2 and *d* = 160 µm from S3. From the figure, it is clear that the behaviour of the different cases depends only on Stk. A unified behaviour of the collection efficiency, impingement velocity, and impingement angle is observed, where droplets of different diameters, exposed to different free-stream velocities and impinging on airfoils of different sizes, exhibit the same behaviour provided they have the same Stokes number. In addition, in the limiting cases of very small Stokes numbers below 0.01, as well as very large Stokes numbers above 5, the results become independent of the Stokes number. In particular, when Stk<0.01, ⟨β⟩ is practically zero. On the other hand, ⟨β⟩, $\left\langle V_{\text{imp}}^{*}\right\rangle$ and ⟨Θ⟩ differ very little for values of the Stokes number that exceed approximately 5.

**Figure 11.**
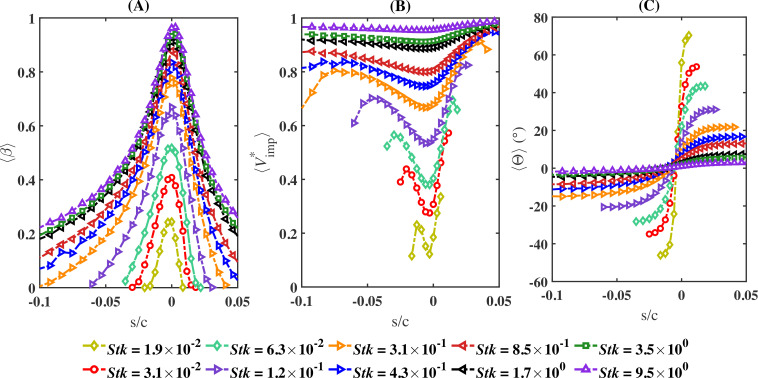
Variation of the collection efficiency (⟨β⟩), normalized impingement velocity ($\left\langle V_{\text{imp}}^{*}\right\rangle$) and impingement angle (⟨Θ⟩) for several Stokes numbers versus the scaled curvilinear position (*s*/*c*) on the airfoil surface.

**Figure 12.**
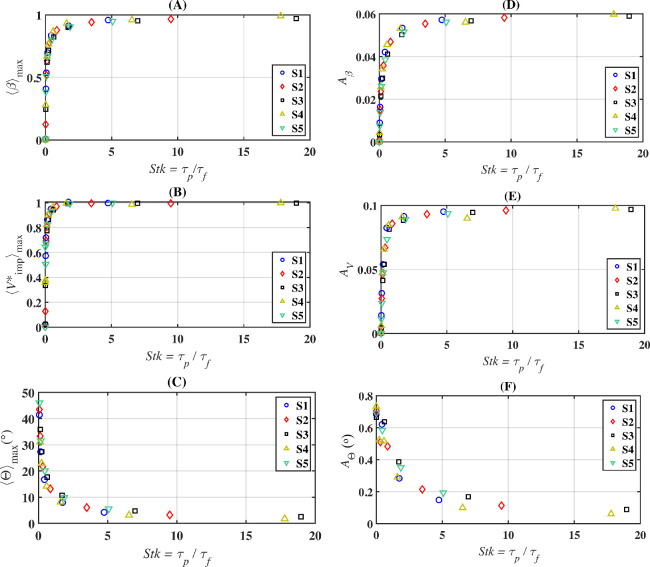
Trends of the maximum collection efficiency ($\langle \beta \rangle_{\text{max}}$), maximum normalized impingement velocity ($\langle V_{\text{imp}}^{*}\rangle_{\text{max}}$) and maximum impingement angle ($\langle \Theta \rangle_{\text{max}}$) versus the Stokes number (panels A, B and C), and trends of the bounded areas in β-s/c, $V_{\text{imp}}^{*}$-s/c and Θ-s/c graphs versus the Stokes number (panels D, E and F)

In figure 11, we show the dependence of the collection efficiency, impingement velocity and impingement angle over a wide range of the Stokes number. The role of the Stokes number as a universal parameter that dictates droplet behaviour is further illustrated in figure 12. This figure consists of six panels arranged in two columns and three rows. The left column displays the maximum values of ⟨β⟩, $\left\langle V_{\text{imp}}^{*}\right\rangle$ and ⟨Θ⟩ versus the Stokes number. The right column shows the area under the curves for ⟨β⟩, $\left\langle V_{\text{imp}}^{*}\right\rangle$ and ⟨|Θ|⟩, as depicted in figure 10, over the range −0.05≤s/c≤0.05, also plotted against the Stokes number. A different marker and colour are used for each case. The areas under each of the respective curves are defined as: $A_{\beta }=\int_{-0.05}^{0.05}\beta d\tilde{s}$, $A_{V}=\int_{-0.05}^{0.05}V_{\text{imp}}^{*}d\tilde{s}$ and $A_{\Theta }=\int_{-0.05}^{0.05}|\Theta |d\tilde{s}$, where $\tilde{s}=s/c$. The data show excellent collapse across all cases from S1 to S5. The maximum values of ⟨β⟩, $\left\langle V_{\text{imp}}^{*}\right\rangle$ and ⟨Θ⟩ are observed to vary sharply from small values of Stk until Stk≈1, after which they level off and saturate. In the case of the collection efficiency, ⟨β⟩ converges to its maximum value $\langle \beta \rangle_{\text{max}}$ of approximately 1 for Stk≳4. This maximum value occurs near the stagnation point. In the case of the impingement velocity, the maximum value approaches the free-stream velocity at Stk≈1.8. As for the maximum impingement angle, it plateaus to a near-zero value beyond Stk≈5. The fact that the maximum impingement velocity matches the free-stream velocity and the impingement angle is near zero further indicates the ballistic nature of those large droplets.

### (f) Validation

**Figure 13.**
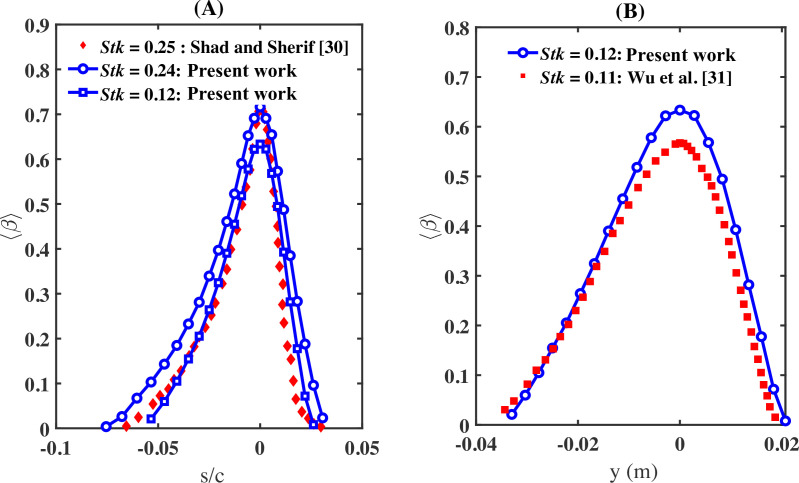
(A) Collection efficiency ⟨β⟩ versus s/c from the present work (blue circles with Stk=0.24 and blue square with Stk=0.12) against the Euler–Euler simulations of [30] having a Stokes number of Stk=0.25. (B) Similar to A but for Stk=0.12 for the present work compared to 0.11 for the Euler–Euler simulations of [31]. In addition, the data are plotted against the vertical coordinate y. We observe a reasonable agreement in both magnitude and range between the Euler–Euler simulations and the present droplet-informed Euler–Lagrange simulations.

We compare the present Euler–Lagrange model against published data. In figure 13A, we plot ⟨β⟩ against s/c for droplets with a Stokes number of Stk=0.24 from case S3 and a Stokes number of Stk=0.12 from case S2 against the data from [30] corresponding to Stk=0.25. In figure 13B, we plot ⟨β⟩ against y for droplets with a Stokes number of Stk=0.12 from case S2 against the data from [31] corresponding to Stk=0.11. The data from both studies [30,31] employ an Euler–Euler approach. Shad & Sherif [30] examined droplets with an MVD of 20 µm, a free-stream velocity of 103 m s^−1^ and an airfoil chord length of 0.53 m at an angle of attack of 4${,}^{\circ }$. Wu *et al.* [31] investigated droplets with an MVD of 16 µm under an incoming Mach number of 0.4 and an angle of attack of 5${,}^{\circ }$, reporting a maximum value of ⟨β⟩=0.58. We observe good acceptable agreement with the published data in both extent and magnitude of the collection efficiency. Some small differences are observed which are due to differences in the numerical approaches.

### (g) Effect of angle of attack (α)

**Figure 14.**
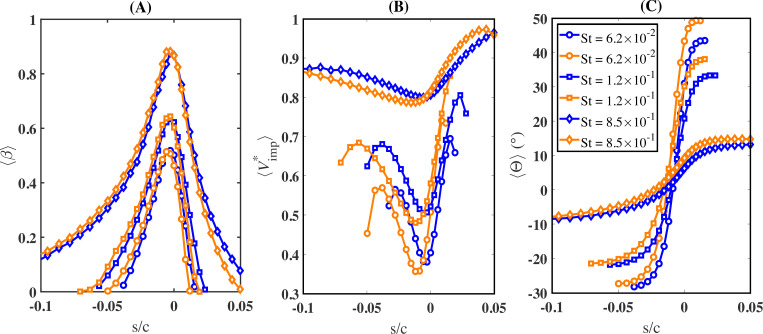
Variation of the collection efficiency (⟨β⟩), normalized impingement velocity ($\left\langle V_{\text{imp}}^{*}\right\rangle$ and impingement angle (⟨Θ⟩) versus the scaled curvilinear position (s/c) on the airfoil surface with respect to the angle of attack. Here, s=0 is defined at the tip of the airfoil. Blue colour represents $\alpha =4^{\circ }$ and orange colour represents $\alpha =6^{\circ }$.

The NACA 0012 is a symmetric airfoil with a stall angle of approximately $15^{\circ }$ [32]. In this section, we consider the effect of the angle of attack by replicating case S2 while increasing the angle of attack α to $6^{\circ }$. In figure 14, and to facilitate comparison between the different angles of attack, we shift the s/c axis such that s=0 now corresponds to the front-most point of the airfoil, which corresponds to the tip of the airfoil along its axis of symmetry. We compare ⟨β⟩, $\left\langle V_{\text{imp}}^{*}\right\rangle$ and ⟨Θ⟩ from cases S2 and S6 for *d* = 13, 29 and 48 µm, corresponding to Stk=0.062,0.12 and 0.85, respectively. The location of the stagnation point at $\alpha =4^{\circ }$ is s=−2.8 mm, while for $\alpha =6^{\circ }$, this location is shifted downward to s=−11.9 mm, resulting in a displacement of 9.1 mm along the curvilinear coordinate on the airfoil. In the case of the collection efficiency, we observe for $\alpha =6^{\circ }$ an increase in ⟨β⟩ for s<0 and a corresponding decrease for s>0. As the airfoil is rotated clockwise, more of its lower side (and less of its upper side) is exposed to the incoming flow. This explains the increased collection efficiency on the lower side (and the decreased collection efficiency on the upper side) of the airfoil. This behaviour is more pronounced for the larger droplets but is observed for all droplet sizes. A similar pattern was reported by Hu *et al.* [33], where an Eulerian approach was used to study the effect of the angle of attack on the droplet collection efficiency.

As for the impingement velocity, we generally observe a decrease in the magnitude of the colliding droplets on the lower side of the airfoil and a corresponding increase on the upper side. This behaviour can be explained as follows: as the angle of attack increases, the deviation in the below-stagnation streamlines must correspondingly increase. This includes a deviation in the streamline direction as well as a reduction in flow velocity magnitude. These changes now occur farther away from the airfoil, which allows the droplets longer time to adjust to the reduced flow velocity, which in turn results in slower impingement velocities. On the other hand, the opposite behaviour occurs on the upper side of the airfoil, such that the flow streamlines have smaller deviations that also occur closer to the airfoil. As such, this results in the increased impingement velocities observed in panel B of figure 14. From panel B, the minimum impingement velocity ($\langle V_{\text{imp}}^{*}\rangle_{\text{min}}$) for all three values of the Stokes number decreases as the angle of attack increases, while the maximum impingement velocity ($\langle V_{\text{imp}}^{*}\rangle_{\text{max}}$) shows the opposite trend. For instance, $\langle V_{\text{imp}}^{*}\rangle_{\text{min}}$ decreases from 0.5 to 0.48 for $\text{Stk}=1.2\times 10^{-1}$, while $\langle V_{\text{imp}}^{*}\rangle_{\text{max}}$ increases from 0.8 to 0.85.

On the other hand, we observe that the impingement angle increases with the angle of attack. For the largest droplets, which follow nearly ballistic trajectories, the increase in the average impingement angle, ⟨Θ⟩, corresponds directly to the two-degree difference in the angle of attack. This occurs because the trajectories of larger droplets are largely unaffected by variations in the flow field. In contrast, smaller droplets are strongly influenced by the flow field, leading to a more pronounced change in the impingement angle for these droplets. From panel C in figure 14, we observe that the maximum impingement angle ($\langle \Theta \rangle_{\text{max}}$), which occurs on the upper side of the airfoil, increases from $33.3^{\circ }$ to $38^{\circ }$ and from $13.2^{\circ }$ to $14.7^{\circ }$ for $\text{Stk}=1.2\times 10^{-1}\;\text{and}\;8.5\times 10^{-1}$, respectively, with an increase in the angle of attack. This shows that droplets with a smaller size (or lower Stk values) are more influenced by the change in the flow due to the change in the angle of attack.

## 4. Conclusions

We conducted three-dimensional, Euler–Lagrange simulations of a droplet-laden airflow impinging on a NACA 0012 airfoil under a variety of operating conditions. We considered free-stream velocities in the range of 60≤U≤240 m s^–1^, chord lengths in the range of 0.5≤c≤2 m and droplet diameters in the range 1≤d≤160 microns. Simulations were run at angles of attack of $\alpha =4^{\circ }$ and $6^{\circ }$. We monitored the droplet collection efficiency ⟨β⟩, droplet normalized impingement velocity $\left\langle V_{\text{imp}}^{*}\right\rangle$ and impingement angle ⟨Θ⟩. We observed that ⟨β⟩ and $\left\langle V_{\text{imp}}^{*}\right\rangle$ increase with U and d, but decrease with airfoil size c. On the other hand, we observed that ⟨Θ⟩ decreases with U and d, but increases with c.

At a fixed angle of attack, ⟨β⟩, $\left\langle V_{\text{imp}}^{*}\right\rangle$ and ⟨Θ⟩ were shown to depend strongly on U, c and d. However, the results for ⟨β⟩, $\left\langle V_{\text{imp}}^{*}\right\rangle$ and ⟨Θ⟩ showed excellent collapse for all cases considered against the global Stokes number Stk (see equation (3.5) for definition). More specifically, we observed that ⟨β⟩ and $\left\langle V_{\text{imp}}^{*}\right\rangle$ increase with increasing Stk, while ⟨Θ⟩ decreases with Stk. In addition, the range over which $\left\langle V_{\text{imp}}^{*}\right\rangle$ and ⟨Θ⟩ vary along the airfoil surface decreases with Stk.

Moreover, we observed that the minimum droplet size required for collision can simply be estimated by calculating the potential flow around the airfoil in terms of the velocity gradient along the stagnation streamline. Our findings showed that only those droplets whose stagnation-streamline Stokes number exceeds the critical value (${\text{Stk}}_{\text{cr}}=0.25$) impinge on the airfoil. We also observed the droplet behaviour to become Stokes-independent for very small and very large values of the Stokes number. For Stokes numbers below approximately 0.01, we observed a near-zero likelihood of impingement, while for Stokes numbers above approximately 5, we observed little variation in droplet behaviour in terms of ⟨β⟩, $\left\langle V_{\text{imp}}^{*}\right\rangle$ and ⟨Θ⟩, i.e. droplet behaviour becomes largely Stokes-independent.

Irrespective of droplet diameter, free-stream conditions and airfoil size, we observed the maximum collection efficiency to plateau to approximately 50% for droplets with large Stokes numbers. Correspondingly, we observed the impingement velocities for these large droplets to approach the free-stream velocity and the impingement angle to remain at approximately 0${,}^{\circ }$ indicating a ballistic trajectory.

While the collection efficiency was observed to reach a maximum value near the airfoil stagnation point, the impingement velocity attained its lowest value at that point. This was attributed to the fact that the stagnation streamline exhibits variations in both velocity magnitude and orientation farther away from the airfoil compared to other streamlines. The farther the variations occur, the more time droplets have to adjust to the slower flow velocities, resulting in slower impingement velocity magnitudes.

The effect of the angle of attack on droplet-impingement characteristics can be summarized as follows: first, in terms of the collection efficiency, β, the changes are minimal for the two-degree shift. We did, however, observe, as the angle of attack increase, β to increase on the lower surface of the airfoil and to correspondingly decrease on the upper surface. This behaviour is true for large and small droplets alike. We also observed the maximum value of β as well as the overall value to remain approximately the same. Second, regarding the impingement velocity, $\left\langle V_{\text{imp}}^{*}\right\rangle$, we noticed a decrease in the magnitude of colliding droplets on the lower side of the airfoil and a corresponding increase on the upper side. As the angle of attack increases, the deviation of the flow streamlines is steeper and occurs farther upstream. As the flow velocity drops, so does the impingement velocity. On the other hand, the flow streamlines along the upper side of the airfoil are relatively less perturbed with the larger angle of attack, leading to larger impingement velocities. Third, for the impingement angle, ⟨Θ⟩, we observed an increase with increasing the angle of attack, with more pronounced effects on smaller droplets. The larger droplets with near ballistic trajectories retain a near horizontal path and as such the impingement angle differs by the change in the angle of attack, which in this case is two degrees. On the other hand, the smaller droplets were found to be more influenced by the flow and as such experienced larger variations.

## Data Availability

All data are contained within the paper.
